# Supplementary material for: Breynia cernua: Chemical Profiling of Volatile Compounds in the Stem Extract and Its Antioxidant, Antibacterial, Antiplasmodial and Anticancer Activity In Vitro and In Silico
Source: Metabolites. 2023 Feb 15;13(2):281. doi: 10.3390/metabo13020281 (PMC9966293; doi:10.3390/metabo13020281)
Supplement: Supplementary file 1 [file metabolites-13-00281-s001.zip › metabolites-2205777-supplementary.pdf]

Supplementary Data

Breynia cernua: Chemical Profiling of Volatile Compounds in the Stem Extract and Its Antioxidant, Antibacterial, Antiplasmodial, and Anticancer Activity In Vitro and In Silico

Hesti Lina Wiraswati [1,2,3,4,*], Nisa Fauziah [1,2,3], Gita Widya Pradini [1,3], Dikdik Kurnia [5], Reza Abdul Kodir [6], Afiat Berbudi [1,2,3], Annisa Retno Arimdayu [4], Amila Laelalugina [4], Supandi [7,8] and Ilma Fauziah Ma'ruf [4,9]

1 Department of Biomedical Sciences, Faculty of Medicine, Universitas Padjadjaran, Sumedang 45363, Indonesia
2 Advance Biomedical Laboratory, Faculty of Medicine, Universitas Padjadjaran, Bandung 40161, Indonesia
3 Infection Working Group, Faculty of Medicine, Universitas Padjadjaran, Sumedang 45363, Indonesia
4 Oncology and Stem Cell Working Group, Faculty of Medicine, Universitas Padjadjaran, Bandung 40161, Indonesia
5 Departement of Chemistry, Faculty of Mathematics and Natural Sciences , Universitas Padjadjaran, Sumedang 45363, Indonesia
6 Pharmacy Study Program, Faculty of Mathematics and Natural Sciences, Universitas Islam Bandung, Bandung 40116, Indonesia
7 PT. Borneo Indobara, Central Jakarta 10350, Indonesia
8 Department of Mining Engineering, Faculty of Technology Mineral, Institut Teknologi Nasional Yogyakarta, Sleman 55281, Indonesia
9 Biochemistry Research Group, Department of Chemistry, Faculty of Mathematics and Natural Sciences, Institut Teknologi Bandung, Bandung 40132, Indonesia
* Correspondence: hesti.lina@unpad.ac.id

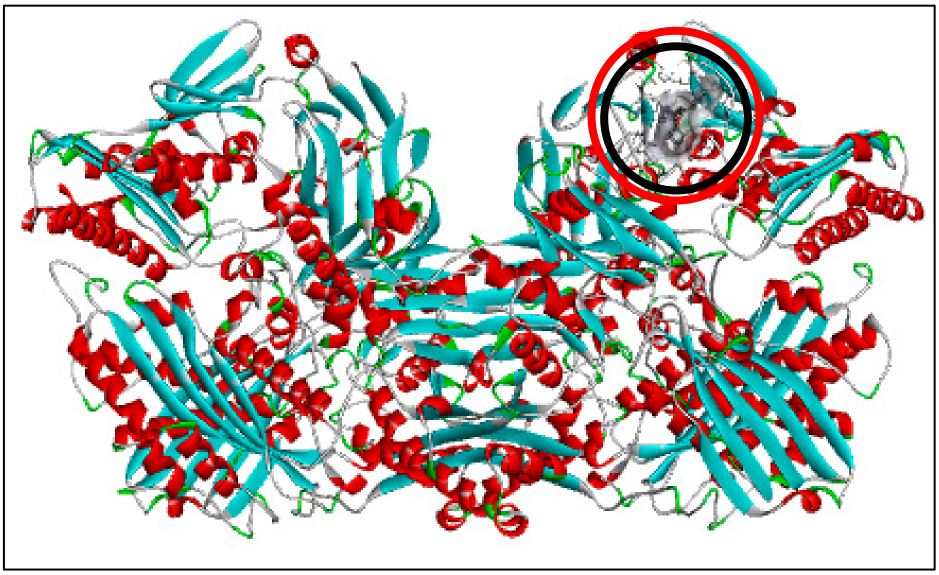

(a)

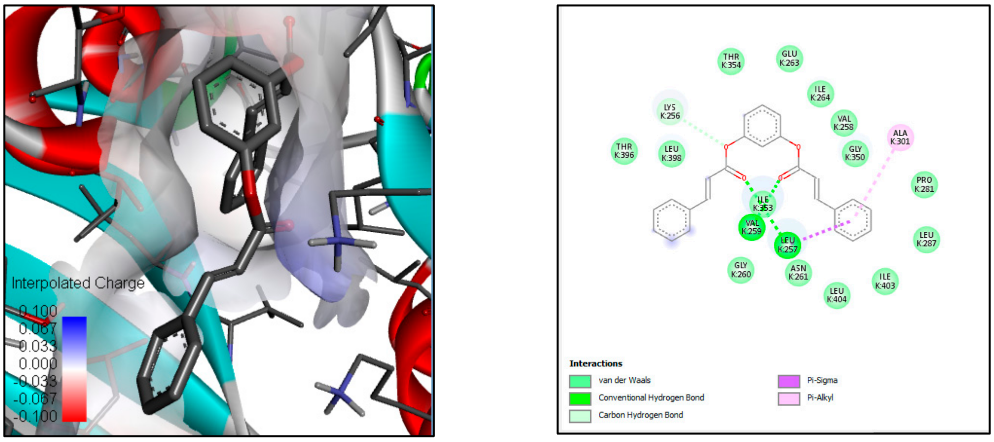

(**b**) (**c**)

Figure S1. Binding interaction of1,3-Phenylene, bis(3-phenylpropenoate) compound with antioxidant target protein (3EUB) based on the binding energy generated by PyRx program.A: (**a**) close-up view of ligand binding on 3EUB (binding site of native ligand and docked ligand were indicated by red and black circle respectively); (**b**) 3D diagram of ligand-protein binding pose; (**c**) 2D interaction of ligand-protein

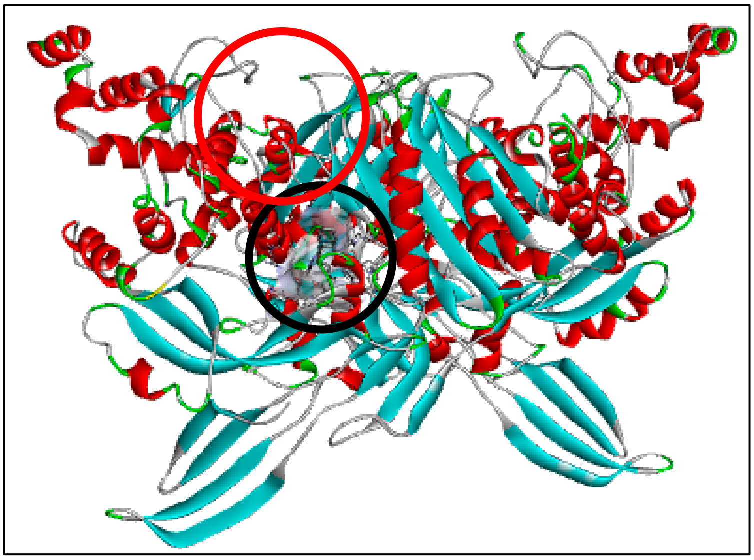

(**a**)

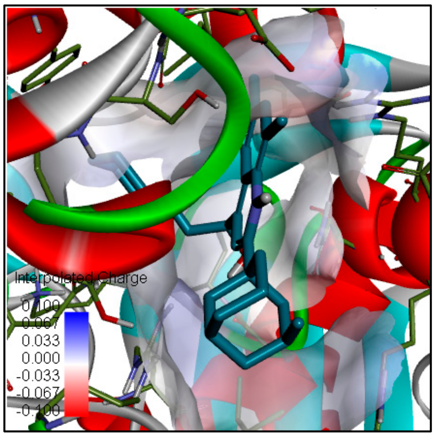

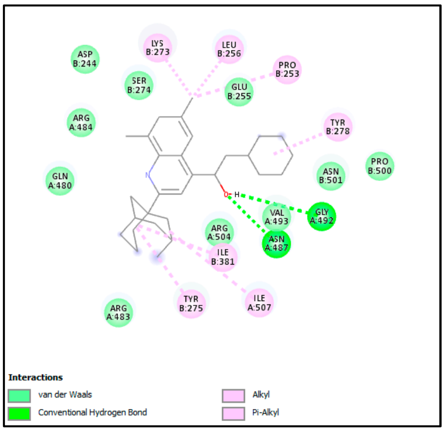

(**b**) (**c**)

Figure S2. Binding interaction of N-[.beta.-Hydroxy-.beta.-[4-[1-adamantyl-6,8-dichloro]quinolyl]ethyl] piperidine compound with antibacterial target protein (3VSL) based on the binding energy generated by PyRx program: (**a**) a close-up view of ligand binding on 3VSL (binding site of native ligand and docked ligand were indicated by red and black circle respectively); (**b**) 3D diagram of ligand-protein binding pose; (**c**) 2D interaction of ligand-protein

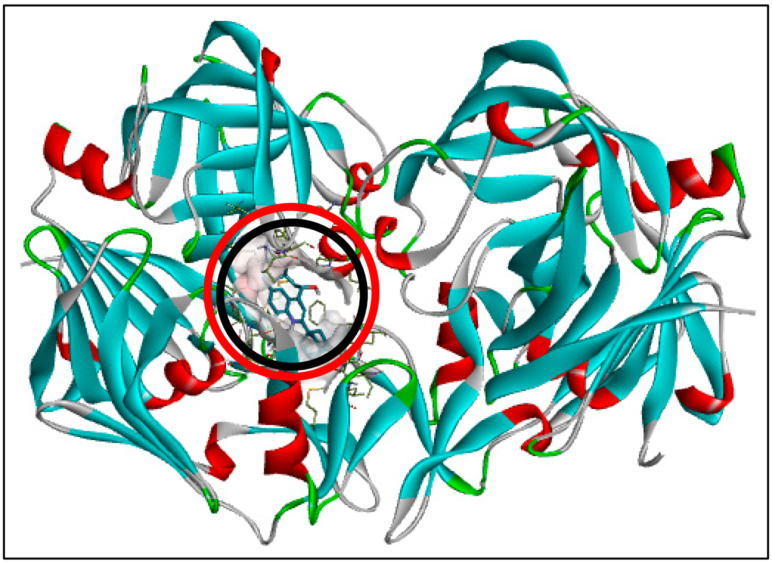

(**a**)

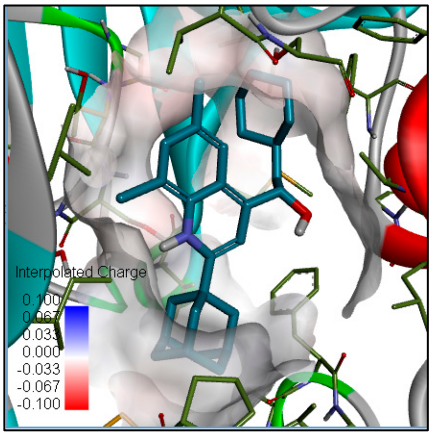

(**b**)

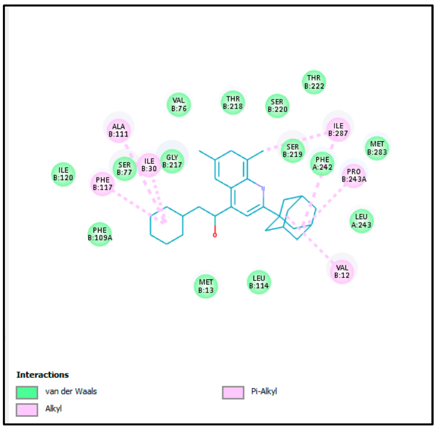

(**c**)

Figure S3. Binding interaction of N-[.beta.-Hydroxy-.beta.-[4-[1-adamantyl-6,8-dichloro]quinolyl]ethyl]
piperidine compound with antiplasmodial target protein (3QS1) based on the binding energy generated by PyRx program: (**a**) a close-up view of ligand binding on 3QS1 (binding site of native ligand and docked ligand were indicated by red and black circle respectively); (**b**) 3D diagram of ligand-protein binding pose; (**c**) 2D interaction of ligand-protein

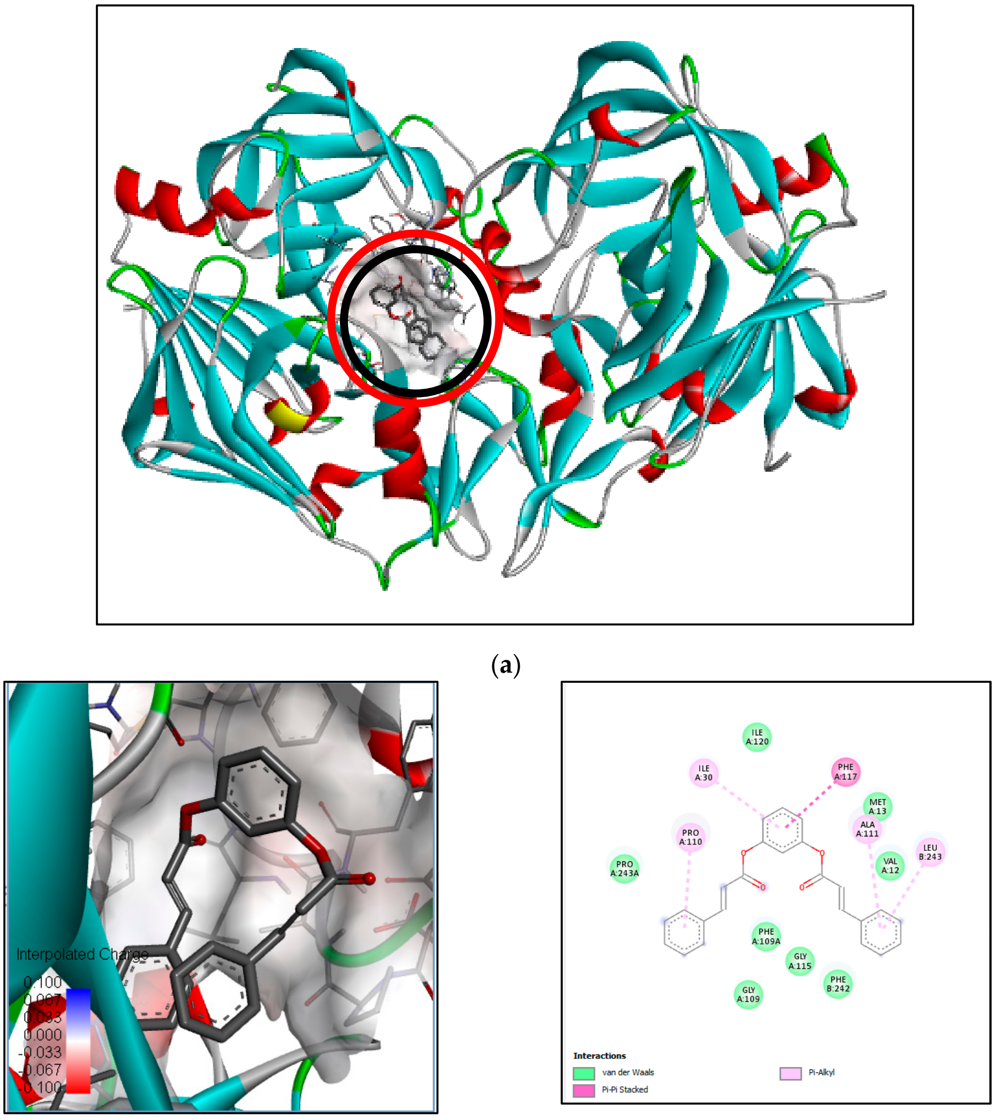

(a)

(b) (c)

Figure S4. Binding interaction of1,3-Phenylene, bis(3-phenylpropenoate) compound with antiplasmodial target protein (3QS1) based on the binding energy generated by PyRx program: (**a**) a close-up view of ligand binding on 3QS1 (binding site of native ligand and docked ligand were indicated by red and black circle respectively); (**b**) 3D diagram of ligand-protein binding pose; (**c**) 2D interaction of ligand-protein

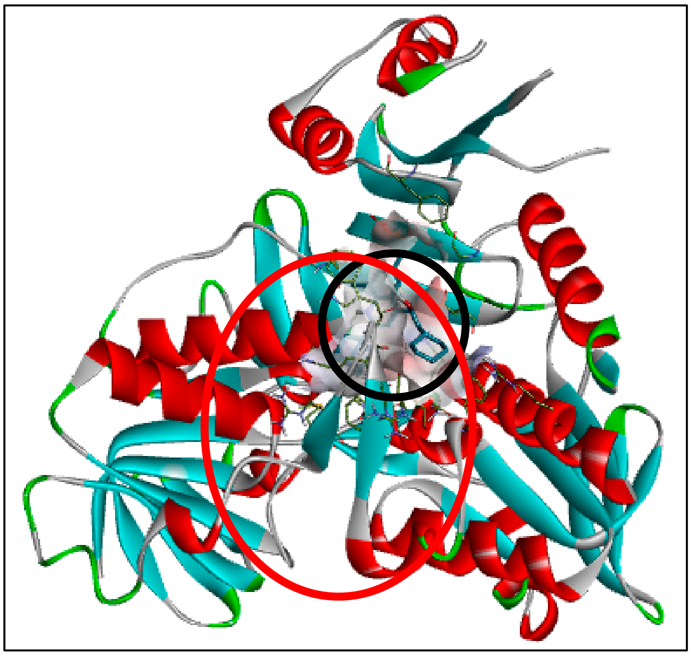

(**a**)

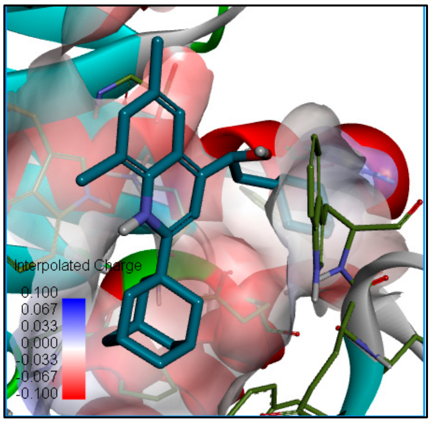

(**b**)

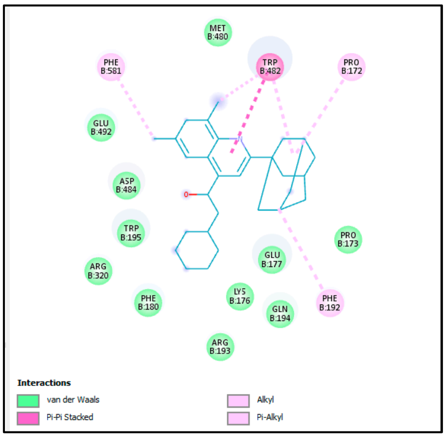

(**c**)

Figure S5. Binding interaction of N-[.beta.-Hydroxy-.beta.-[4-[1-adamantyl-6,8-di-chloro]quinolyl]ethyl]
piperidine compound with anticancer target protein (3GD4) based on the binding energy generated by PyRx program: (**a**) a close-up view of ligand binding on 3GD4 (binding site of native ligand and docked ligand were indicated by red and black circle respectively); (**b**) 3D diagram of ligand-protein binding pose; (**c**) 2D interaction of ligand-protein

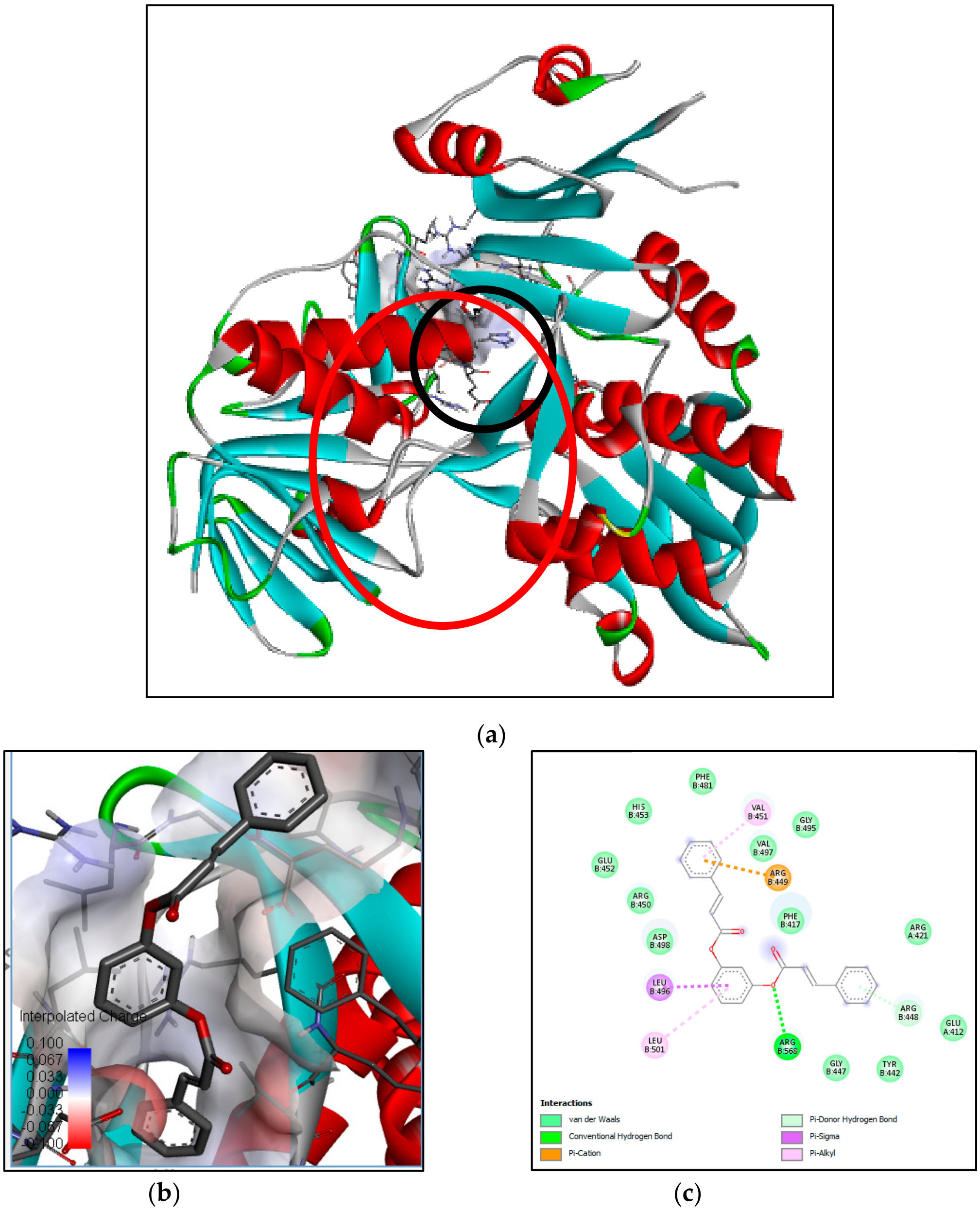

Figure S6. Binding interaction of 1,3-Phenylene, bis(3-phenylpropenoate) compound with anti-cancer target protein (3GD4) based on the binding energy generated by PyRx program: (**a**) a close-up view of ligand binding on 3GD4 (binding site of native ligand and docked ligand were indicated by red and black circle respectively); (**b**) 3D diagram of ligand-protein binding pose; (**c**). 2D interaction of ligand-protein

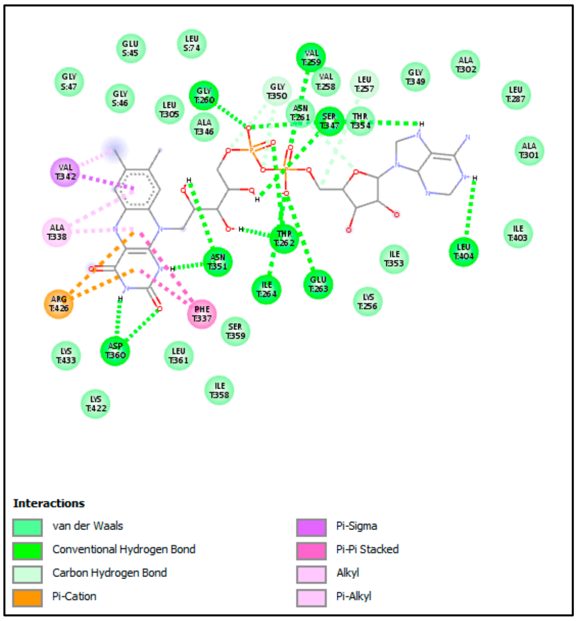

(a)

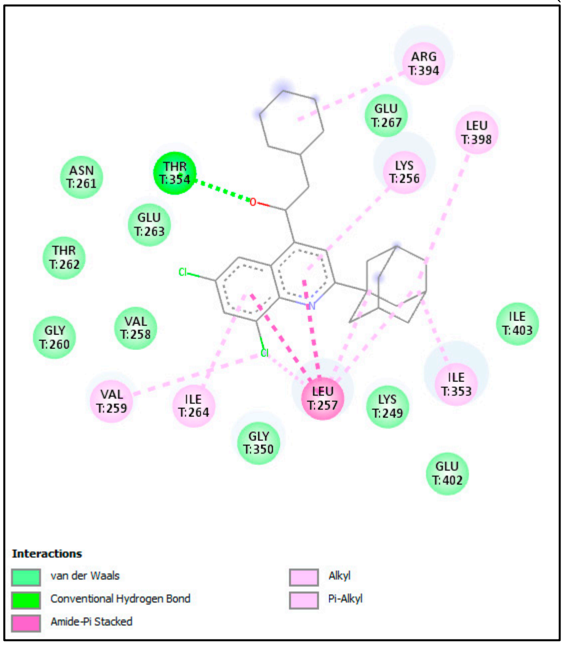

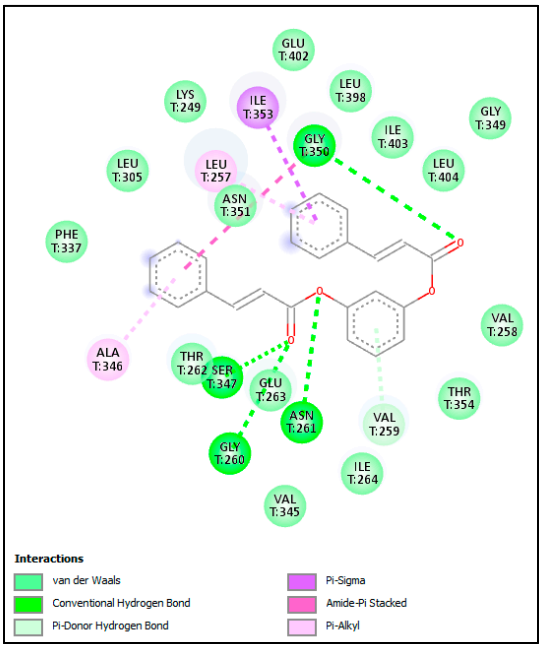

(b) (c)

Figure S7. 2D interaction of Antioxidant protein target (3EUB) and ligands: (**a**) FLAVIN ADENINE DINUCLEOTIDE (native ligand); (**b**) N-[.beta.-Hydroxy-.beta.-[4-[1-adamantyl-6,8-di-chloro]quinolyl]ethyl]piperidine; (**c**) 1,3-Phenylene, bis(3-phenylpropenoate)

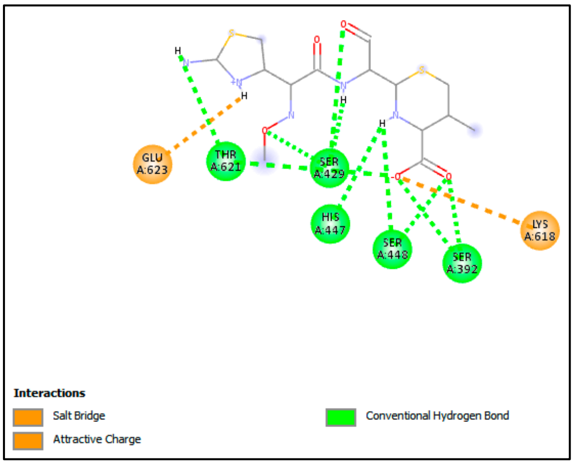

(**a**)

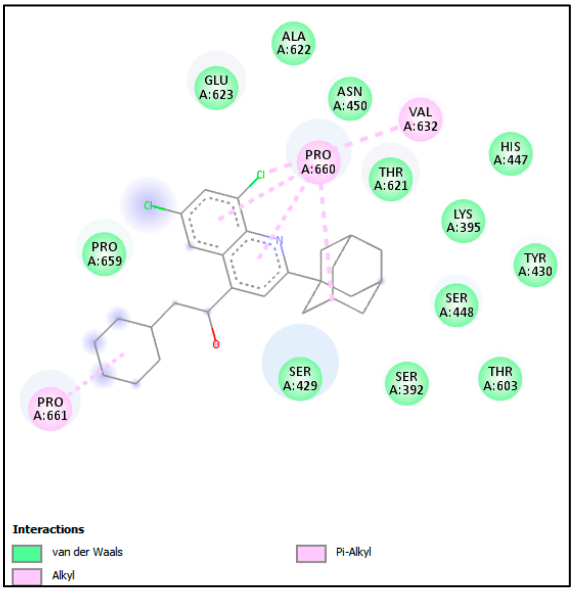

(**b**)

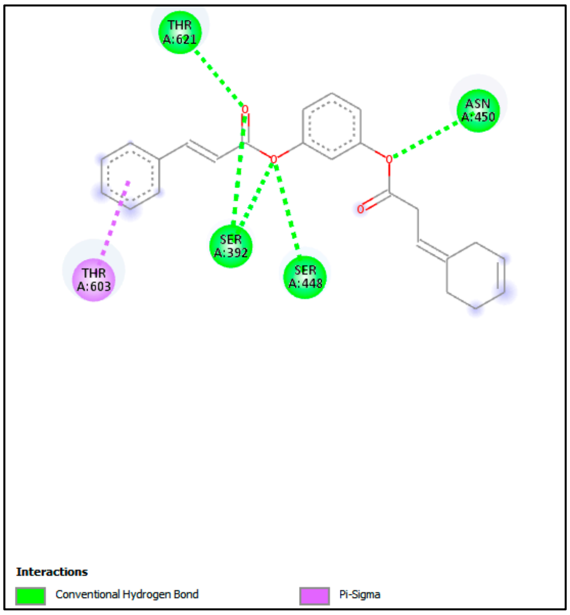

(**c**)

Figure S8. 2D interaction of Antibacterial protein target (3VSL) and ligands: (**a**) CEFOTAXIME (native ligand) (**b**) N-[.beta.-Hydroxy-.beta.-[4-[1-adamantyl-6,8-dichloro]quinolyl]ethyl]piperidine; (**c**) 1,3-Phenylene, bis(3-phenylpropenoate)

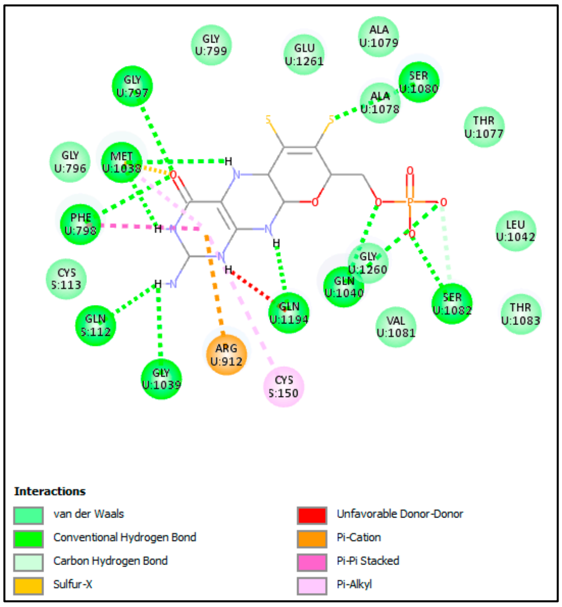

(a)

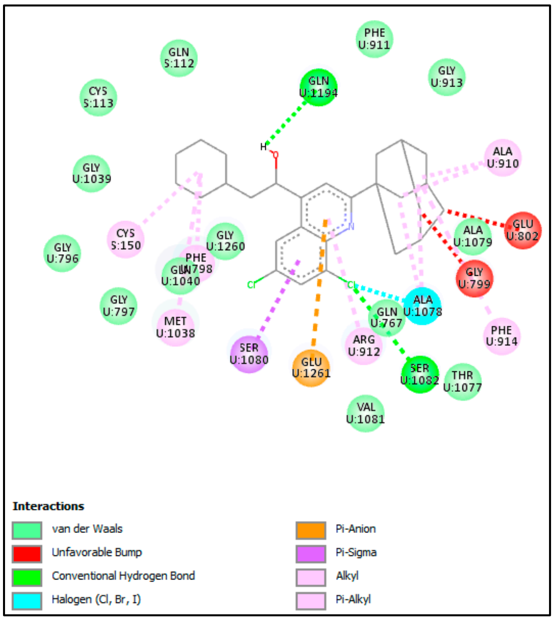

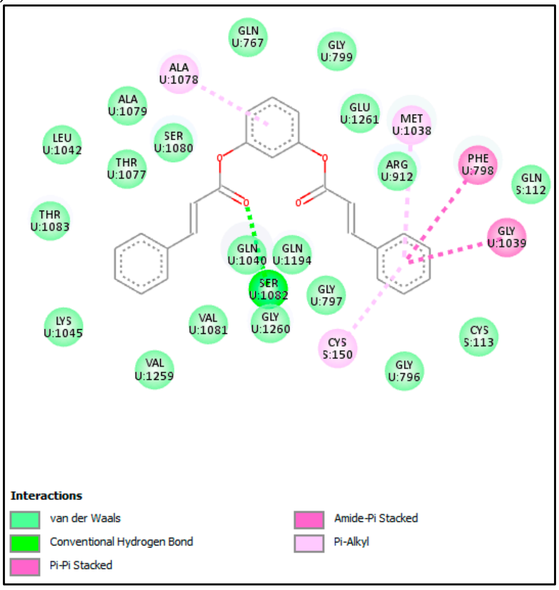

(b) (c)

Figure S9. 2D interaction of Antioxidant protein target (3EUB) and ligands: (**a**) PHOSPHONIC ACIDMONO-(2-AMINO-5,6-DIMERCAPTO-4-OXO-3,7,8A,9,10,10A-HEXAHYDRO-4H-8-OXA-1,3,9,10-TETRAAZA-ANTHRACEN-7-YLMETHYL)ESTER (native ligand); (**b**) N-[.beta.-Hydroxy-.beta.-[4-[1-adamantyl-6,8-dichloro]quinolyl]ethyl]piperidine; (**c**) 1,3-Phenylene, bis(3-phenylpropenoate)

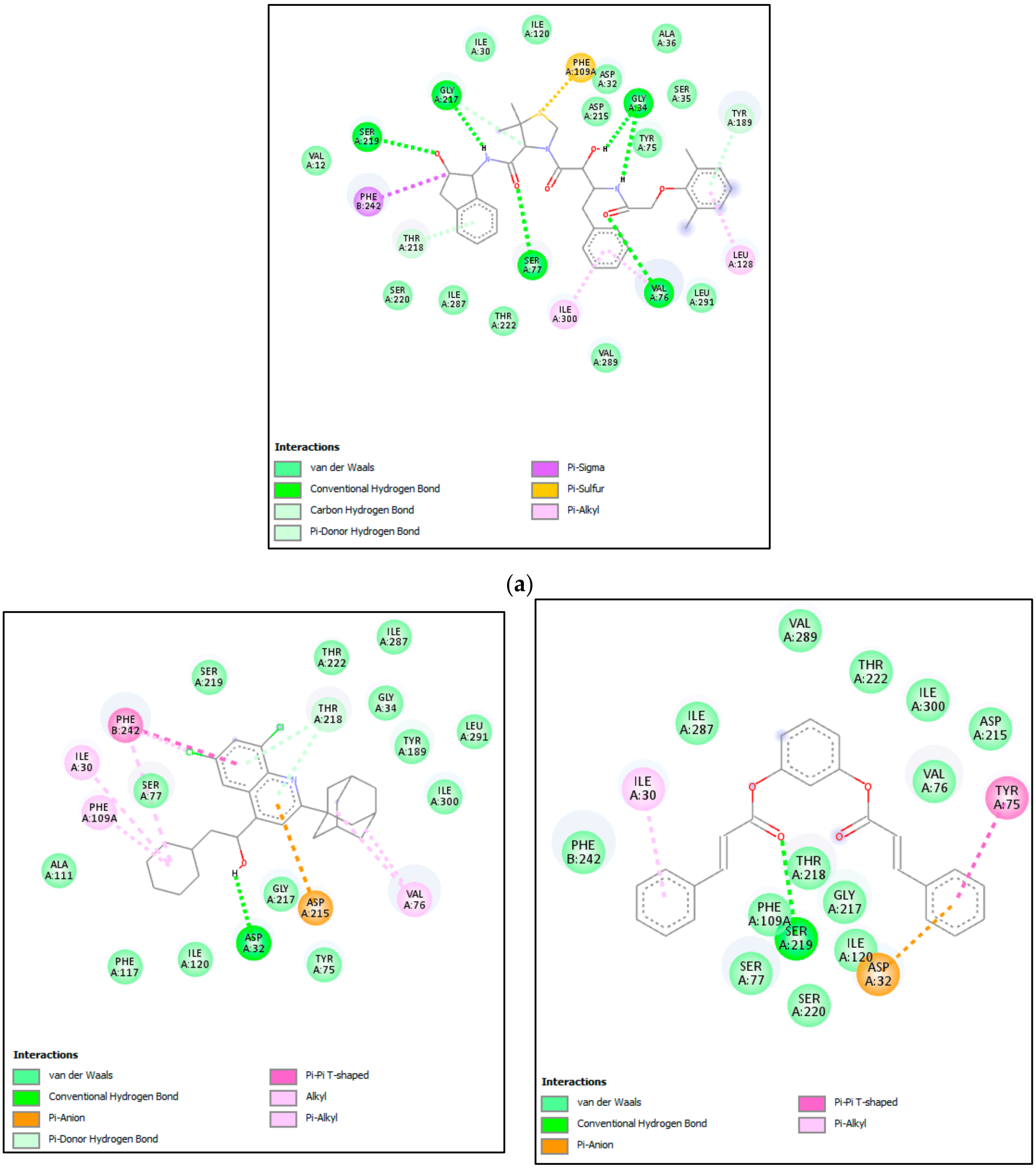

(**a**)

(**b**) (**c**)

Figure S10. 2D interaction of Antiplasmodial protein target (3QS1) and ligands: (**a**) (4R)-3-[(2S,3S)-3-{[(2,6-dimethylphenoxy)acetyl]amino}-2-hydroxy-4-phenylbutanoyl]-N-[(1S,2R)-2-hydroxy-2,3-dihydro-1H-inden-1-yl]-5,5-dimethyl-1,3-thiazolidine-4-carboxamide (native ligand); (**b**) N-[.beta.-Hydroxy-.beta.-[4-[1-adamantyl-6,8-dichloro]quinolyl]ethyl]piperidine; (**c**) 1,3-Phenylene, bis(3-phenylpropenoate)

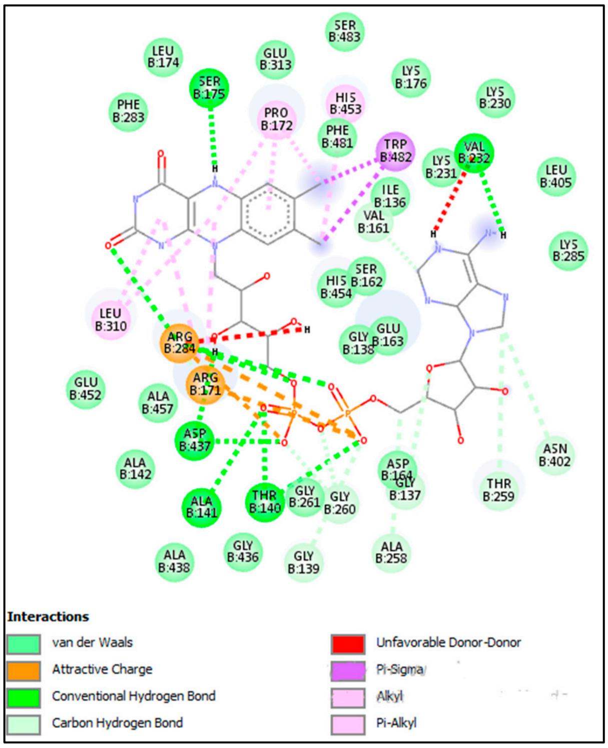

(**a**)

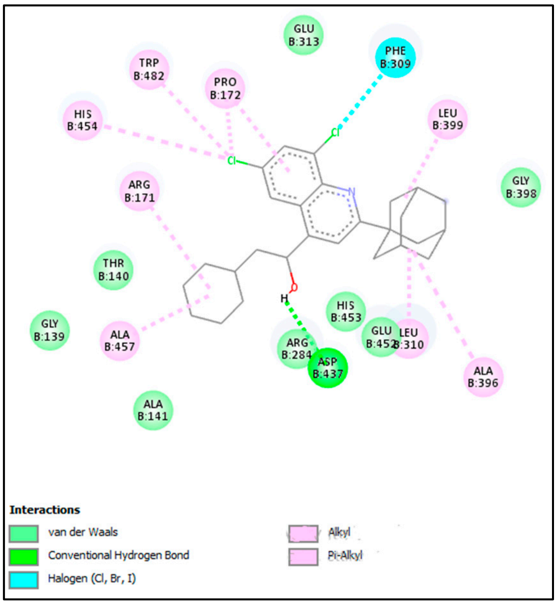

(**b**)

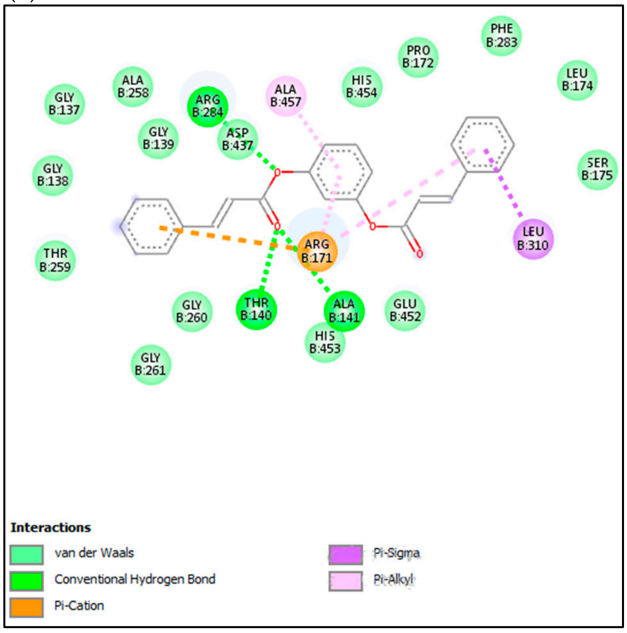

(**c**)

Figure S11. 2D interaction of Anticancer protein target (3GD4) and ligands; (**a**) FLAVIN ADENINE DINUCLEOTIDE (native ligand); (**b**) N-[.beta.-Hydroxy-.beta.-[4-[1-adamantyl-6,8-di-chloro]quinolyl]ethyl]piperidine; (**c**) 1,3-Phenylene, bis(3-phenylpropenoate)

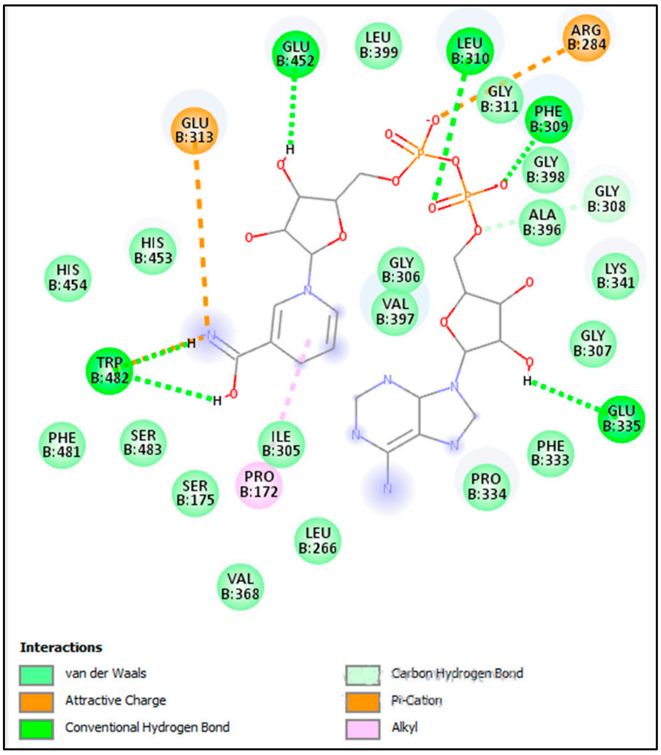

(**a**)

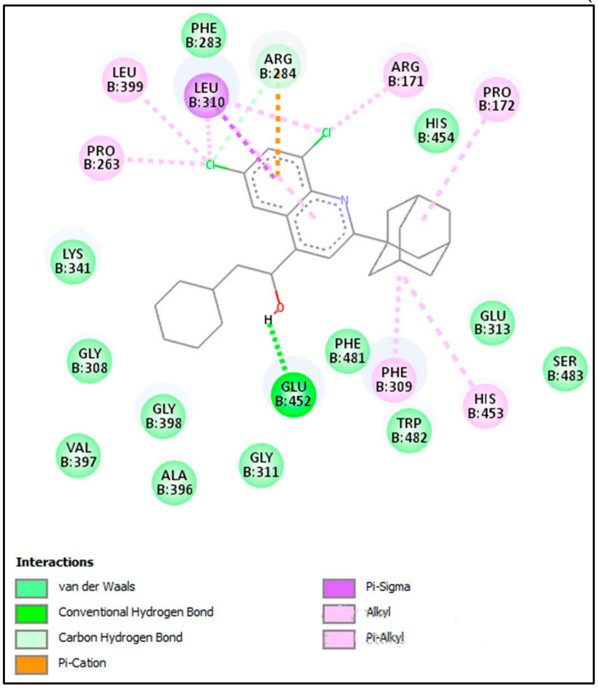

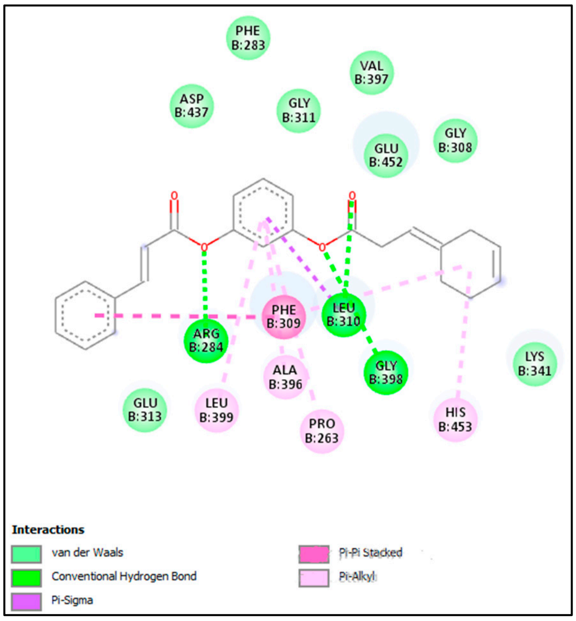

(**b**) (**c**)

Figure S12. 2D interaction of Anticancer protein target (3GD4) and ligands: (**a**) NICOTINAMIDE-ADENINE-DINUCLEOTIDE (native ligand); (**b**) N-[.beta.-Hydroxy-.beta.-[4-[1-adamantyl-6,8-di-chloro]quinolyl]ethyl]piperidine; (**c**) 1,3-Phenylene, bis(3-phenylpropenoate)
